# The Novel Extracellular Cyclophilin A (CyPA) - Inhibitor MM284 Reduces Myocardial Inflammation and Remodeling in a Mouse Model of Troponin I -Induced Myocarditis

**DOI:** 10.1371/journal.pone.0124606

**Published:** 2015-04-20

**Authors:** David Heinzmann, Anna Bangert, Anna-Maria Müller, Saskia N. I. von Ungern-Sternberg, Frederic Emschermann, Tanja Schönberger, Madhumita Chatterjee, Andreas F. Mack, Karin Klingel, Reinhard Kandolf, Miroslav Malesevic, Oliver Borst, Meinrad Gawaz, Harald F. Langer, Hugo Katus, Gunter Fischer, Andreas E. May, Ziya Kaya, Peter Seizer

**Affiliations:** 1 Medizinische Klinik III, Kardiologie und Kreislauferkrankungen, Eberhard Karls Universität Tübingen, Tübingen, Germany; 2 Department of Internal Medicine III, University of Heidelberg, Heidelberg, Germany; 3 Institute of Anatomy, Eberhard Karls Universität Tübingen, Tübingen, Germany; 4 Institute for Pathology and Neuropathology, Department of Molecular Pathology, Eberhard Karls Universität Tübingen, Tübingen, Germany; 5 Martin-Luther-Universität Halle-Wittenberg, Institut für Biochemie, Abteilung Enzymologie, Projektgruppe gFP5, Halle (Saale), Germany; 6 Max-Planck-Institut für Biophysikalische Chemie Göttingen, BO Halle (Saale), Göttingen, Germany; Albert Einstein College of Medicine, UNITED STATES

## Abstract

Cyclophilins are a group of highly conserved cytosolic enzymes that have a peptidylprolyl cis/trans isomerase activity. Cyclophilin A (CyPA) can be secreted in the extracellular space by inflammatory cells and upon cell death. The presence of CyPA in patients with non-ischemic cardiomyopathy is associated with poor clinical prognosis. Here, we investigated the inhibition of extracellular CyPA in a mouse model of troponin I-induced autoimmune myocarditis using the strictly extracellular CyPA-inhibitor MM284. Since A/J mice develop severe inflammation and fibrosis after immunization with murine cardiac troponin I (mcTn I), we used this model to analyze the effects of an extracellular CyPA inhibition. As extracellular CyPA-inhibitor we used the recently described CsA-derivate MM284. *In vitro* studies confirmed that MM284 inhibits CyPA-induced monocytic migration and adhesion. A/J mice immunized with mcTnI were treated with MM284 or vehicle every second day. After 28 days, we found a considerable reduction of myocardial injury and fibrosis. Further analysis revealed a reduced myocardial presence of T-cells and macrophages compared to control treated animals. Whereas MMP-9 expression was reduced significantly by MM284, we observed no significant reduction of inflammatory cytokines such as IL-6 or TNFα. Extracellular CyPA plays an important role in autoimmune myocarditis for myocardial damage and fibrosis. Our data suggest a new pharmacological approach for the treatment of myocardial inflammation and reduction of cardiac fibrosis by inhibition of extracellular CyPA.

## Introduction

Inflammatory cardiomyopathy is a major cause of severe heart failure and heart transplantation [1]. To date, no effective causal therapy is available [2]. Cyclophilins (CyPs) are a group of highly conserved cytosolic enzymes that have a peptidylprolyl cis/trans isomerase activity [3]. It has been shown that Cyclophilin A (CyPA) is involved in various pathophysiological mechanisms of cardiovascular diseases [4–6]. CyPA can be secreted in the extracellular space by inflammatory stimuli and is released upon cell death [7, 8]. Especially the interaction of extracellular CyPA and the Extracellular Matrix Metalloproteinase Inducer (EMMPRIN, CD147) has been identified as an important factor in inflammatory processes such as leucocyte chemotaxis and induction of matrix metalloproteinases (MMP) [3, 6].

We have recently shown that enhanced expression of CyPA is associated with inflammatory cardiomyopathy [9]. In addition, CyPA expression predicts poor prognosis in patients with non-ischemic cardiomyopathy [10]. Inhibition of intra- and extracellular CyPA in a model of coxsackievirus B3-induced myocarditis resulted in strong reduction of myocardial inflammation and fibrosis *in vivo* [11]. However, myocardial damage was not affected significantly since inhibition of CyPA led to an impaired recruitment of T-cells and thus, a reduced virus clearance [11].

Intracellular CyPA is involved in cell signaling, calcium homoeostasis and transport mechanisms [3, 12]. Many side effects of CyPA-inhibitors such as cyclosporin A could be ascribed to the inhibition of intracellular CyPs and to calcineurin inhibition via gain-of-function [13, 14]. Thus, the use of unrestricted CyPA-inhibitors is problematic if applied in humans.

Recently, a novel group of CyPA-inhibitors has been described targeting only extracellular CyPA functions [15–17]. MM284 is such a modification of cyclosporin A, which is restricted to the extracellular space [16]. It therefore offers a new possibility to investigate the significance and therapeutic potential of inhibition of extracellular CyPA in pathophysiological conditions.

More than 50% of patients with non-ischemic cardiomyopathy show signs of inflammation. Moreover, the role of extracellular CyPA in the context of non-pathogen associated inflammatory cardiomyopathy has not been investigated, so far [10].

In this study, we analyzed the effects of extracellular CyPA-inhibition using the novel extracellular CyPA-inhibitor MM284 *in vitro* and in a model of non pathogen-induced autoimmune myocarditis.

## Materials and Methods

### Monocyte migration and adhesion to endothelium

Monocytes were isolated from healthy donors as described before [6]. A modified 48-well Boyden chamber (Neuro Probe, Gaithersburg, MD) was used for migration studies. The upper chambers with 20.000 monocytes in 50μl media were separated by a filter with 5μm pores from the lower chambers. The lower chambers contained media supplemented with CyPA (200nM, R&D Systems, Minneapolis, MN, USA), MM284 (200; 500; 800nM) or SDF-1α (50ng/ml, R&D Systems) as positive control. All tests were conducted with cells from 5 different donors using triplicates for each well. After 4h at 37°C, cells adherent to the lower filter surface were visualized by May-Grünwald/Giemsa staining and counted. A chemotactic index of migrated cells was calculated using the negative control as base level.

A flow chamber assay was performed as previously described [6]. In brief, gelatin-coated coverslips covered with confluent human umbilical vein endothelial cells (HUVEC, FC-0003, obtained from CellSystems, Troisdorf, Germany) were treated with TNFα (50ng/ml, Peprotech, Hamburg, Germany) and IFNγ (20ng/ml, Peprotech) for 4h to activate endothelial cells. Then, isolated monocytes were stimulated with CyPA (200nM, R&D Systems) or preincubated with CyPA (200nM) and MM284 (500nM) overnight. Subsequently, monocytes (200.000/ml) were perfused over the activated endothelial cells with shear rates of 2000s^-1^. All experiments were recorded in real time for offline evaluation.

### Induction and treatment of autoimmune myocarditis

For the induction of autoimmune myocarditis, A/J mice (Harlan Winkelmann GmbH, Borchen, Germany) were injected subcutaneously with murine cardiac troponin I (mcTnI) in complete Freund’s adjuvant at day 0, 7 and 14, as previously described [18]. Mice were treated with MM284 (10mg/kg bodyweight) or vehicle by intraperitoneal injection from day 0 to day 28 every second day. After 28 days mice were sacrificed and the hearts were collected for further analysis. Animals were bred and kept under specific pathogen-free conditions and received humane care at the animal facilities of the University of Heidelberg.

### Echocardiography and histological analysis of mice

For echocardiography, mice were examined 28 days after induction of autoimmune myocarditis using a Vevo 2100 small animal imaging system (VisualSonics, Amsterdam, The Netherlands), as previously described [6].

For histological analysis, Hematoxylin and Eosin (H&E) as well as Masson’s Trichrome staining was performed using standard staining protocols and reagents. For immunohistochemical staining of murine paraffin fixed cardiac sections, antibodies against Mac-3 (1:200, rat, clone M3/84, BD Pharmingen, Heidelberg, Germany), CD3 (1:300, rabbit, clone SP-7, Thermo Fisher Scientific, Fremont, CA, USA), and CyPA (1:100, rabbit, AB42408, Abcam, Cambridge, UK) were used. For secondary detection single step HRP-Polymer detection kits (rat-on-mouse and rabbit-on-rodent HRP-Polymer kits, both Biocare Medical, Concord, CA, USA) and HistoGreen HRP-substrate kit (Linaris, Dossenheim, Germany), as well as Streptavidin/HRP and DAB (both Dako, Glostrup, Denmark) were used. Sections stained with H&E as well as Masson’s Trichrome were analyzed using a scoring system based on relative area of inflammation in the myocardium and fibrosis, respectively (grade 0: no infiltration / fibrosis, grade 1: infiltration / fibrosis in up to 5% of the cardiac section, grade 2: infiltration / fibrosis in 6–10%, grade 3: infiltration / fibrosis in 11–30%, grade 4: infiltration / fibrosis in 31–50%, grade 5: infiltration / fibrosis in more than 50%) [18]. Analysis was performed in a blinded manner. Sections stained for Mac-3 and CD3 were analyzed using a scoring system (grade 0: no infiltration of Mac-3 / CD3 positive cells, up to grade 4: strong infiltration of Mac-3 / CD3 positive cells) in a blinded manner.

For immunofluorescence images, paraffin fixed sections from mice 28 days after induction of experimentally induced myocarditis were stained using anti-CyPA antibody (goat, AF3589, R&D Systems), Alexa fluor 488 conjugated secondary antibody (1:150, rabbit, Life technologies, Eugene, OR, USA), rhodamine phalloidin (1:300, Life technologies); staining the actin cytoskeleton, and To-Pro-3 iodine (1:1000, Life technologies). Confocal laser scanning images were acquired using a Zeiss LSM5 EXCITER Confocal Laser Scanning Microscope (Carl Zeiss Micro Imaging, Jena, Germany) with a 40x oil immersion objective.

### Real time PCR analysis

Frozen murine heart tissue samples were homogenized with TRIzol reagent (Life Technologies, Carlsbad, CA, USA) using a rotor-stator system. Subsequent RNA isolation was performed as specified by the manufacturer. For synthesis of cDNA the Transcriptor First Strand cDNA Synthesis Kit from Roche (Roche Diagnostics, Mannheim, Germany) was used. For RT-PCR, the Mesa Fast qPCR Master Mix Plus for SYBR assay (Eurogentec, Seraing, Belgium) was used along with primers for IL-6, TNFα, MMP-9. The following primer sequences were used for IL-6 fw: ctc tgg gaa atc gtg gaa at; rev: cca gtt tgg tag cat cca tc, for TNFα fw: atg aga agt tcc caa atg gc; rev: ctc cac ttg gtg gtt tgc t, and for MMP-9 fw: cca aag acc tga aaa cct cca a; rev: cgg ccc ggg tgt aac c. Assays were quantified using a LightCycler 480 System (Roche Diagnostics) with the following protocol: 95°C for 5min followed by 40 cycles of 95°C for 15sec and 60°C for 60sec. Results were analyzed using the LightCycler Ver. 1.5 software with the Ct advanced relative quantification function.

### MM284 permeability assay

To evaluate the membrane permeability of MM284, a previously described competition assay was used [17]. In brief, 2x10^5^ THP1 cells (DSMZ, Braunschweig, Germany) on poly-lysine coated cover slips were incubated with 1mM of Fluo-mCsA (a cell permeable CsA derivate with a fluorescent dye, MM466 [17]) alone, Fluo-mCsA + 100μM MM284, or Fluo-mCsA + 100μM NIM811 (a cell permeable CsA derivate, kind gift from Novartis) for 2h. Displacement of intracellular Fluo-mCsA by MM284 or NIM811 was evaluated using confocal laser scanning microscopy (LSM5 EXCITER Confocal Laser Scanning Microscope, Carl Zeiss Micro Imaging, Jena, Germany). Nuclei were stained using To-Pro-3 iodine (Life technologies).

### Ethics statement

All experiments were conducted in strict accordance to the German animal protection law. The protocol was approved by the Committee on the Ethics of Animal Experiments of the University of Heidelberg (Registration Number. G-68/09) and complied with the institution's guidelines.

### Statistical analysis

For statistical analyses Student’s t-test and Mann-Whitney U test was applied using SPSS 21 (IBM, Armonk, NY, USA) and Graphpad 6.0 (Graphpad Software, La Jolla, CA, USA). p < 0.05 was considered as statistically significant.

## Results

### Extracellular CyPA is associated with cardiac fibrosis in autoimmune myocarditis

**Fig 1:**
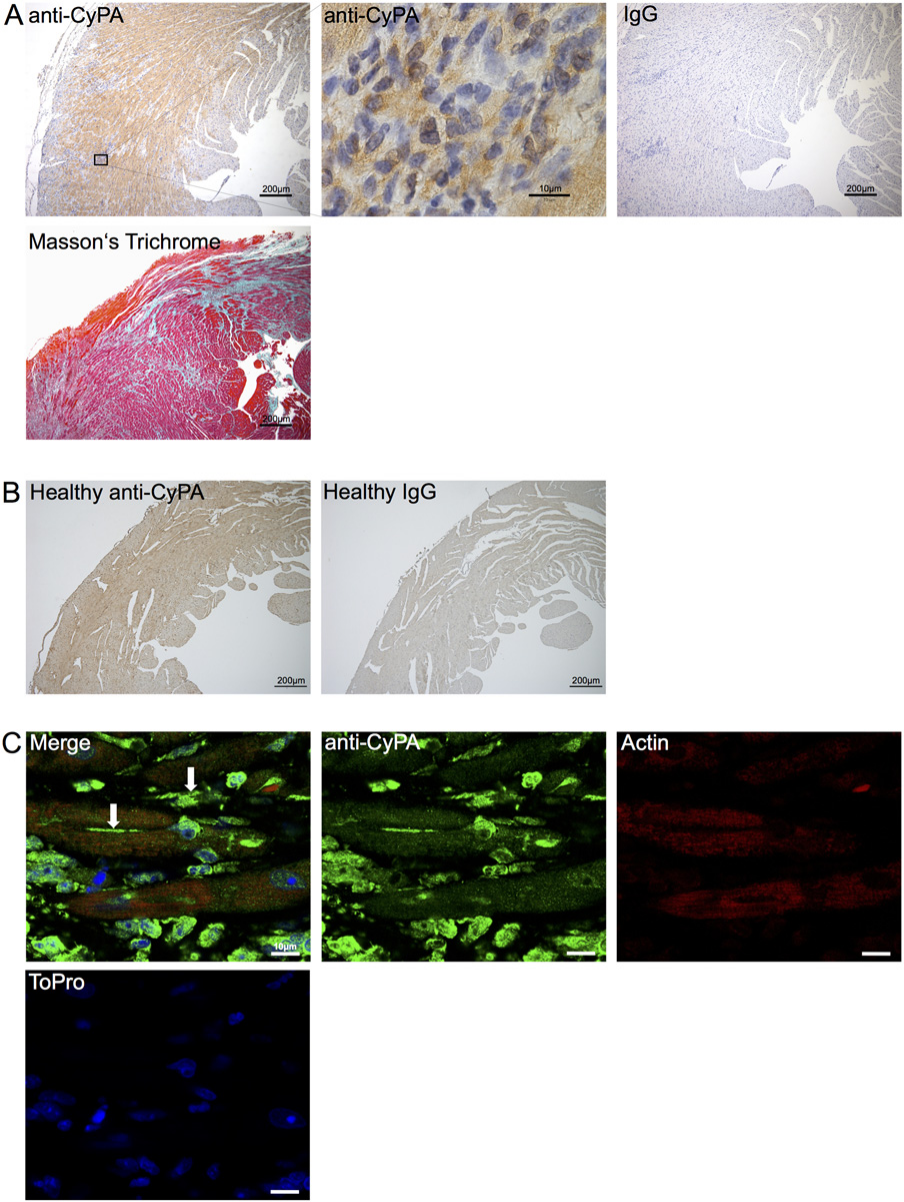
Extracellular Cyclophilin A is associated with myocardial fibrosis. **A**, Representative stainings of heart sections from mice 28 days after induction of troponin I-induced autoimmune myocarditis. Sections were stained with Masson’s Trichrome, anti-CyPA, and IgG-control as indicated. Marked area is magnified in the middle panel. **B**, Representative image of healthy control mice stained with anti-CyPA or IgG control. **C**, Myocardial stainings from mice 28 days after induction of troponin I-induced autoimmune myocarditis. Myocardial sections were stained with anti-CyPA (green), rhodamin phalloidin (red) for actin cytoskeleton, and ToPro-3 for nuclei (blue), as described in materials and methods. Arrows indicate localization of CyPA in the extracellular space.

A/J mice develop severe cardiac fibrosis associated with increased expression of CyPA upon immunization with troponin I (Fig 1A) compared to healthy control mice (Fig 1B) [18, 19]. Confocal microscopy revealed further evidence that in autoimmune myocarditis CyPA is not only present intracellularly, but can be also detected in the extracellular space (Fig 1C). Thus, we decided to use this model to study the effects of an inhibition of extracellular CyPA on myocardial fibrosis and inflammation.

### MM284 inhibits CyPA-induced migration and adhesion of monocytes

**Fig 2:**
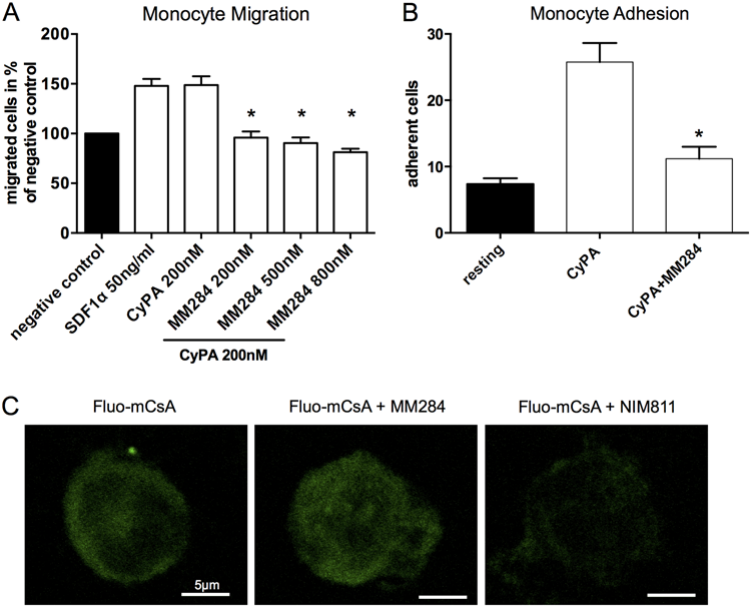
Extracellular Cyclophilin A—inhibition by MM284 attenuates migration of human monocytes *in vitro*. **A**, Effects of MM284 on Cyclophilin A-induced chemotaxis were studied using a modified Boyden chamber. Migration of human monocytes was assessed using media, SDF-1α (50ng/ml) as positive control, CyPA (200nM), or CyPA (200nM) + MM284 (200; 500; and 800nM). Cells were incubated for 4h at 37°C. Migrated cells were counted and a chemotactic index was calculated (n = 5). **B**, Adhesion of monocytes under flow conditions to activated human umbilical vein endothelial cells. Data are shown as mean ± SEM. **C**, Cell membrane permeability of MM284 was assessed using a competition assay. MM284 or NIM811 (a cell permeable CsA derivate) was used to displace Fluo-mCsA (a fluorescently labeled (green), cell permeable CsA derivate) from the cytoplasm of THP1 cells. The presence of Fluo-mCsA in the cytoplasm after treatment was assessed using confocal laser scanning microscopy. * indicates p < 0.05 compared to CyPA 200nM.

First, we analyzed the inhibitory potential of the novel extracellular CyPA-inhibitor MM284 on monocyte migration and adhesion after stimulation with recombinant CyPA. MM284 diminished CyPA-induced migration in a concentration dependent manner (Fig 2A). Additionally, treatment with MM284 reduced CyPA-induced monocyte adhesion to HUVEC under flow conditions as well (Fig 2B).

To provide further evidence that MM284 acts only extracellularly compared to cell permeable CsA derivates such as NIM811 [20], we analyzed a competition assay using fluorescently labeled Cyclosporin A (Fluo-mCsA). Whereas NIM811 could replace the binding of Fluo-mCsA to intracellular CyPA (as seen by a reduction of fluorescence intensity), MM284 showed no relevant replacement of Fluo-mCsA (Fig 2C).

### MM284 reduces myocardial damage and fibrosis in troponin I—induced autoimmune myocarditis

**Fig 3:**
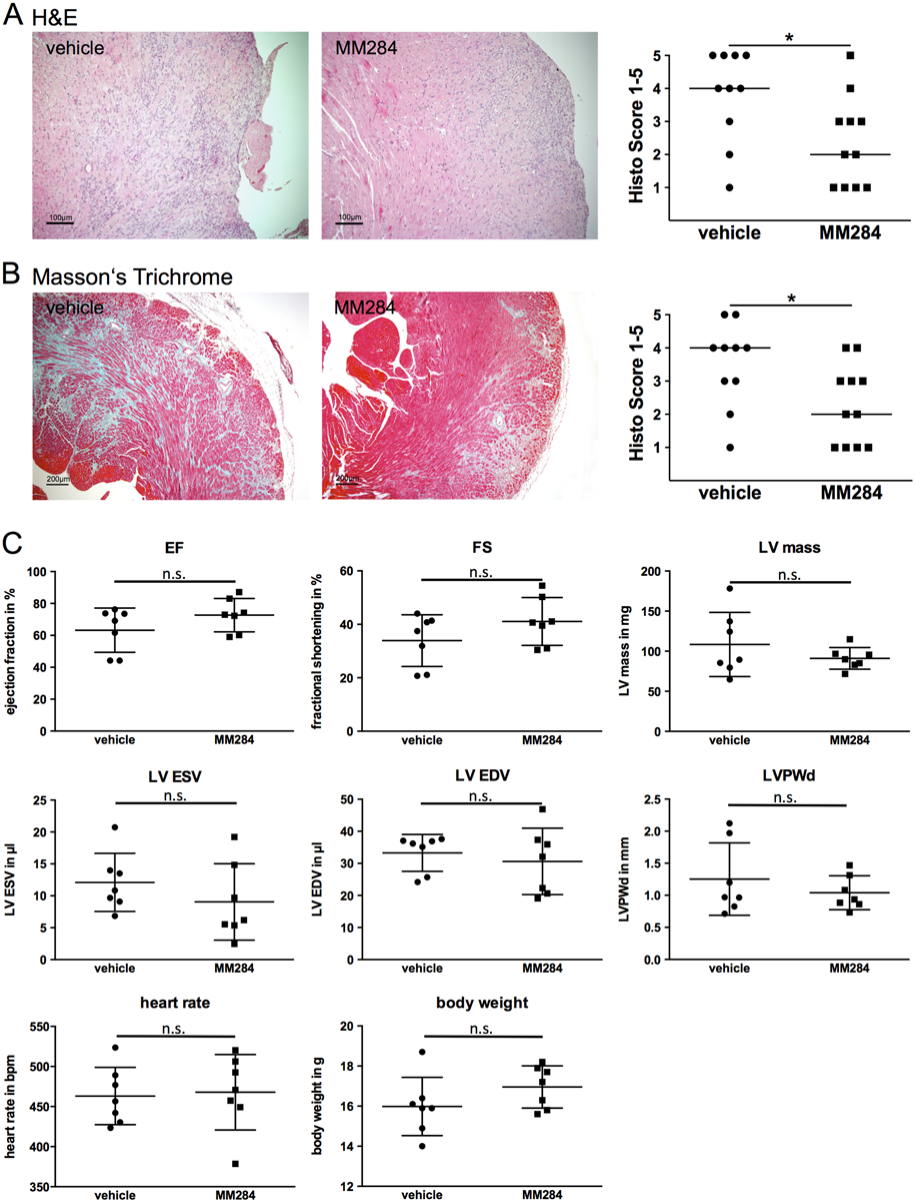
MM284 reduces cardiac inflammation and fibrosis in autoimmune myocarditis in mice. Troponin I-induced autoimmune myocarditis was studied in A/J mice. **A**, Hematoxylin and Eosin staining (H&E) was used to determine the area of cardiac damage 28 days after induction of myocarditis. **B**, Masson’s Trichrome staining was used to determine the extent of fibrotic remodeling in the myocardium 28 days after induction of experimental autoimmune myocarditis. Representative images for each treatment are shown. Data in the right panels show individual scoring results (n ≥ 10), horizontal bars indicate medians, * indicates p < 0.05. **C**, 28 days after induction of autoimmune myocarditis, small animal echocardiography was used to evaluate left ventricular function (n = 7). Left ventricular ejection fraction (EF), fractional shortening (FS), left ventricular mass (LV Mass), left ventricular end-systolic volume (LV ESV), left ventricular end-diastolic volume (LV EDV), left ventricular posterior wall in diastole (LVPWd), heart rate, and body weight are shown. n.s. indicates not significant.

In the next step we used an experimental model of autoimmune myocarditis in mice for analyzing the effects of an extracellular CyPA-inhibition *in vivo*. Parallel to immunization with troponin I, A/J mice were treated with MM284 or vehicle as control. After 28 days of treatment, mice were sacrificed and the hearts were analyzed for cardiac damage (H&E staining). The damaged area in mice treated with MM284 was markedly reduced compared to the control group (Fig 3A).

Masson’s Trichrome staining of MM284 treated mice showed markedly reduced cardiac fibrosis compared to mice treated with vehicle (Fig 3B). However, small animal echocardiography revealed no significant difference (Fig 3C).

### MM284 reduces myocardial inflammation via inhibition of myocardial T-cell and macrophage recruitment

**Fig 4:**
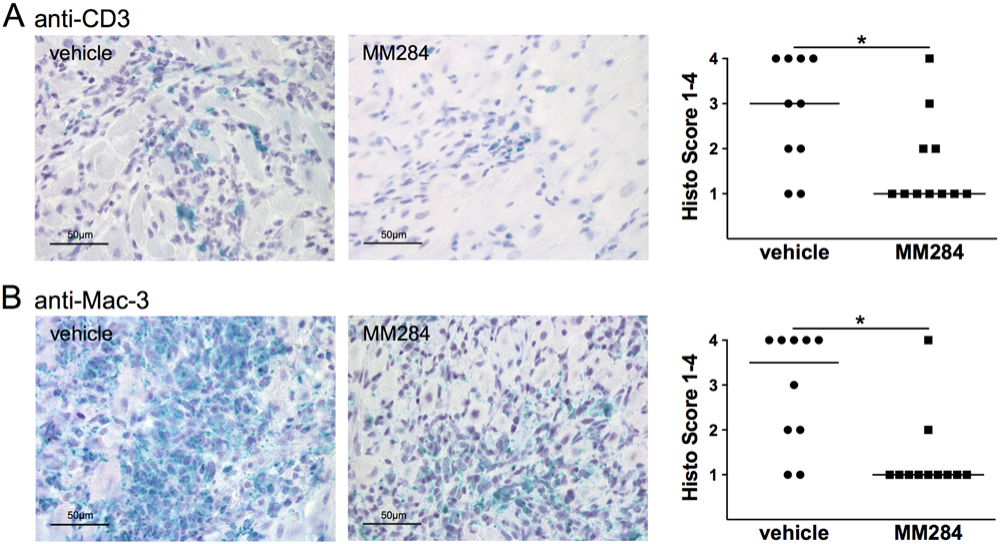
MM284 reduces infiltration of T-cells and macrophages in autoimmune myocarditis in mice. **A**, **B**, Infiltration of T-cells and macrophages was assessed using anti-CD3 and anti-Mac-3 staining. Representative images for each treatment are shown. Data in the right panels show individual scoring results (n ≥ 10), horizontal bars indicate medians, * indicates p < 0.05.

**Fig 5:**
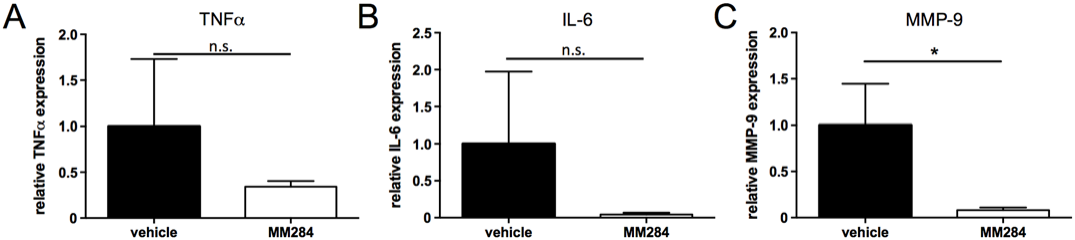
Influence of MM284 on myocardial TNFα, IL-6 and MMP-9 expression. Realtime PCR assay was performed to evaluate expression of TNFα (**A**), IL-6 (**B**) and MMP-9 (**C**) in the myocardium. Data are shown as mean of relative expression ± SD, n ≥ 8. * indicates p < 0.05, n.s. indicates not significant.

To analyze the mechanism by which MM284 influences myocardial damage and fibrosis we investigated the presence of inflammatory cells such as T-cells and macrophages in the myocardium (Fig 4A and 4B). As expected, based on our *in vitro* findings, MM284 reduced recruitment of T-cells and macrophages remarkably. Consistent with these findings, quantitative real time PCR revealed a reduced expression of MMP-9 (Fig 5C), whereas the expression of other proinflammatory cytokines such as TNFα and IL-6 showed no significant differences (Fig 5A and 5B).

## Discussion

The relevance of extracellular CyPA in cardiovascular diseases has been described in various studies for ischemia and reperfusion injury, virus-induced myocarditis, atherosclerosis and aortic aneurysms [4–6].

We have recently shown that the expression of CyPA is enhanced in patients with inflammatory cardiomyopathy and predicts clinical outcome [9, 10]. In the current study we provide evidence that inhibition of extracellular CyPA using the novel CyPA-inhibitor MM284 reduces myocardial inflammation and fibrosis in a mouse model of troponin I-induced autoimmune myocarditis.

Our current data suggest that the poor prognosis associated with increased CyPA expression could be ascribed to increased inflammation and consecutive myocardial fibrosis.

The extracellular CyPA-EMMPRIN interaction has been reported to be important for the recruitment of inflammatory cells. It is furthermore critically involved in the induction of MMPs, which play a pivotal role in myocardial remodeling and fibrosis. In the current study, we show that in a pathogen-free myocarditis model pharmacological inhibition of extracellular CyPA reduces cardiac fibrosis and diminishes cardiac inflammation, accompanied by a reduced recruitment of inflammatory cells such as T-cells and macrophages. Moreover, treatment with MM284 significantly reduces the myocardial expression of MMP-9, which is considered to be one of the key players amongst pro-fibrotic MMPs [21]. However, analysis of echocardiographic data revealed no significant improvement in left ventricular function, which may reach statistical significance on later time points or by analyzing a higher number of animals.

We do not show direct evidence that MM284 binds to extracellular CyPA *in vivo*. However, our *in vitro* findings provide evidence, that MM284 acts strictly extracellularly and that MM284 is a potent inhibitor of extracellular CyPA as shown for CyPA-induced migration and adhesion of monocytes.

It has been reported that other cyclophilins such as CyPB and CyPD are involved in fibrotic processes [22, 23]. These effects are due to intracellular inhibition of these cyclophilins. Nevertheless, we cannot exclude that binding of MM284 to extracellular CyPB may have influenced our results.

Based on our findings it is tempting to speculate that in patients with inflammatory cardiomyopathy treatment with tissue restricted CyPA-inhibitors may attenuate myocardial remodeling.

In conclusion, inhibition of extracellular CyPA seems to be a novel target for the treatment of non-pathogen associated inflammatory cardiomyopathy. Thus, our data may help to develop a new treatment strategy for inflammatory cardiomyopathy without affecting important intracellular functions of CyPA like T-cell activation, calcium homeostasis, and signaling.
